# The Story of the Insane from Year to Year

**Published:** 1904-10-15

**Authors:** 


### THE STORY OF THE INSANE FROM YEAR TO YEAR.

Seventeenth Annual Series.

### LIMERICK DISTRICT ASYLUM.

Here the numbers were almost stationary, being 622 at the beginning of the year, and 626 at the end of it. There were 128 first admissions, 32 readmissions, 62 discharged recovered, 45 discharged relieved, and 46 deaths. The total number under treatment was 773, and the average number resident was 625. The recoveries stand at 38·8 per cent. of the admissions, and the deaths at 7·4 per cent. of the average number resident. Of the year's deaths 9 were caused by cerebral and spinal diseases, and it may be remarked that general paralysis does not appear at all as a cause of death; consumption was the cause in 13, and other lung diseases in 4. Of the year's admissions we find that such moral causes as domestic trouble, mental anxiety and religion were the causes of insanity in 42 cases, and that among the physical causes hereditary influence accounts for 38, previous attacks for 18, and drink for 14. These figures show that about 33 per cent. of the admissions were due to hereditary influence and drink, causes it ought to be possible to eradicate, and which the advent of a higher civilisation and a higher morality may be hoped to do something to lessen if not to prevent. Dr. O'Neill recognises the prevalence of phthisis in asylums, and he remarks on the curiously sudden way in which it often shows itself among the insane. At Limerick, as already pointed out, there were 13 deaths from this disease in a total number of 46 deaths, and this proportion must represent a much higher number of patients afflicted with the disease, therefore we think that some provision should be made for their separate treatment. For this purpose nothing answers better than cheap wooden buildings. Much structural work is at present in progress, and among others the kitchen, laundry, and stores are being reconstructed, and, what must have tended greatly to increase the cheerfulness of the wards, the windows have been lowered. Dr. O'Neill has only one assistant medical officer. The cost per head per annum was £24 14s. 1d.

### LEICESTER AND RUTLAND ASYLUM.

This asylum contained 500 patients at the beginning of the year, and 494 at the end of it. There were 97 first admissions, 30 readmissions, 38 discharged recovered, 19 discharged relieved, 19 discharged not improved, and 57 died. The total number under treatment was 627, and the average number resident was 498. The percentages of recoveries on the admissions were 19·35 among the men, and 40 among the women, giving an average of 29·92 per cent. The deaths were 11·44 per cent. on the average number resident. The recoveries were below the average and the deaths above it. Eighteen of the deaths were caused by cerebral and spinal diseases, 15 by consumption, and 3 by other lung diseases. As moral causes of the insanity in the year's admissions, shock appears once in the returns, mental anxiety three times and domestic trouble once. As physical causes we have intemperance in drink in 8 cases, epilepsy in 9, hereditary influence in 8, and in 11 no cause was ascertained. As there were 15 deaths from tubercular diseases, it is evident that some attempt should be made to separate consumptive patients from the others, but neither the chairman nor the medical superintendent refers to the subject of isolation of such cases. We note that the chief nurse and housekeeper (Mrs. Beaumont) received a pension of £80 a year after 24 years' service, that Attendant Leonard received £30, and Attendant Shill £40. There is a charitable fund attached to this asylum. Last year the subscriptions to this fund amounted to £39 18s., interest and dividends to £560, and £497 10s. was received for maintenance. The average number of patients benefited during the year was 22, and these were received at sums varying from 1s. a week up to 15s. a week, and at least half the cases paid 7s. a week or less. It is easy to see what a good work this charity continues to do, and what a blessing it must be to many patients who would otherwise have had the stigma of pauperism attached to them. The average cost per head per week of the county patients was a fraction over 9s. 5d.

